# Supplementary material for: Two fingerprinting sets for Humulus lupulus based on KASP and microsatellite markers
Source: PLoS One. 2022 Apr 14;17(4):e0257746. doi: 10.1371/journal.pone.0257746 (PMC9009645; doi:10.1371/journal.pone.0257746)
Supplement: Attachment — Submitted filename: Response To Reviewers.docx [file pone.0257746.s019.docx]

Dear Editors,

We would like to thank the editor and the reviewers for through review of this manuscript.

We have done the following:

1. We have removed the financial disclosure from the acknowledgement section
2. We would like the Financial Disclosure to be:

‘We thank USDA CRIS 2072-21000-049-00D and CRIS 2072-21000-051-00D for financial support for this project. We thank the Brewers Association for additional funding of this project. The funders had no role in study design, data collection and analysis, decision to publish, or preparation of the manuscript.’

3. There were no citations in the abstract
4. We deleted “data not shown” in our manuscript since the data we referred to is not a core part of the research being presented.
5. All data for this article is publicly available and was stated clearly in Data Availability
6. We removed lingual errors etc. across the manuscript as you see in the ‘Manuscript with Track Changes’
7. We checked the format requirements at [https://journals.plos.org/plosone/s/file?id=wjVg/PLOSOne_formatting_sample_main_body.pdf](https://gcc02.safelinks.protection.outlook.com/?url=https%3A%2F%2Fjournals.plos.org%2Fplosone%2Fs%2Ffile%3Fid%3DwjVg%2FPLOSOne_formatting_sample_main_body.pdf&data=04%7C01%7C%7C13faca2ac2be4c0ab68808d9a6068d1b%7Ced5b36e701ee4ebc867ee03cfa0d4697%7C0%7C0%7C637723370515810877%7CUnknown%7CTWFpbGZsb3d8eyJWIjoiMC4wLjAwMDAiLCJQIjoiV2luMzIiLCJBTiI6Ik1haWwiLCJXVCI6Mn0%3D%7C2000&sdata=y%2BbpsFrrakL96gQZxXl2bF6xB3mXQGJq%2FmkavRx%2Bf%2Fs%3D&reserved=0)  and changed the format to meet it

Thank you again and we look forward to acceptance of this manuscript.

Best Wishes,

Nahla Bassil
